# Short-term Clinical Outcomes of Unexpected Culture-positive *Cutibacterium acnes* (Formerly *Propionibacterium acnes*) in Open Orthopaedic Surgery

**DOI:** 10.5435/JAAOSGlobal-D-22-00010

**Published:** 2022-07-06

**Authors:** Brent R. Sanderson, Atul Saini, Emerald Chiang, Kristen Linton, Earl W. Brien

**Affiliations:** From the Department of Orthopaedic Surgery, Community Memorial Health System, Ventura (Dr. Sanderson, Dr. Saini) (Sanderson and Saini); the College of Osteopathic Medicine of the Pacific, Western University of Health Sciences, Pomona (Chiang); the California State University Channel Islands, Camarillo, CA (Dr. Linton); the Orthopaedic Oncology in the Samuel Oschin Comprehensive Cancer Institute (Dr. Brien); the Sarcoma and Bone Tumor Program, Los Angeles, CA (Dr. Brien); and the Department of Orthopaedics, Physician Relations and Referral Enhancement (Dr. Brien).

## Abstract

**Introduction:**

The clinical significance and treatment recommendations for an unexpected positive *Cutibacterium acnes* (*C acnes*) culture remain unclear. The purpose of our study was to evaluate the clinical effect of a *C acnes* positive culture in patients undergoing open orthopaedic surgery.

**Methods:**

Patients with a minimum of one positive *C acnes* intraoperative culture were retrospectively reviewed over a 7-year period. True *C acnes* infection was defined as culture isolation from ≥1 specimens in the presence of clinical or laboratory indicators of infection.

**Results:**

Forty-eight patients had a positive intraoperative *C acnes* culture. 4.2% had a *C acnes* monoinfection, and 12.5% of the patients had a coinfection. The remainder was classified as indeterminate. Significant differences were identified between the indeterminate and true *C acnes* infection groups, specifically in patients with surgery history at the surgical site (*P* = 0.04), additional antibiotic therapy before surgery (*P* < 0 .001), and postoperative clinical signs of infection (*P* < 0 .001).

**Discussion:**

Suspicion for true *C acnes* infection should be raised in patients with surgery site history, antibiotic therapy before surgery, and clinical infectious signs. The indeterminate unexpected positive culture patients had a low risk of developing a true clinical infection that required antibiotic therapy.

*Propionibacterium acnes* (*P acnes*) is an anaerobic, slow-growing, non–spore-forming, gram-positive organism part of the normal skin flora and is most notable in orthopaedic literature for its pathogenicity in deep and superficial surgical site infections.^1^ *Cutibacterium acnes (C acnes)* is able to colonize the acidic, anaerobic environment of the dermal sebaceous glands and the epidermis.^2^ Furthermore, *C acnes* can form a resistant biofilm, especially on orthopaedic implants, to elude the human immune response.^3^ Recently, reclassification of *P acnes* to *C acnes* has been suggested on the basis of genomic and metagenomic investigations.^3,4^

Regardless of nomenclature, one positive *C acnes* culture may indicate true infection that is clinically significant or relatively asymptomatic. Postoperative infections after orthopaedic surgery can result in notable long-term sequelae including long-term antibiotic therapy and possible return to the operating room for irrigation and débridement, removal of implants, or revision surgery.^1,3^ Most of the current literature focuses on shoulder surgeries given the proposed increased *C acnes* burden around the shoulder girdle.^5,6,7,8,9,10,11,12^ In the shoulder arthroplasty literature, *C acnes* represents the causative organism in 12% to 51.3% of prosthetic joint infections.^12,13^

The clinical diagnosis and relevance of unexpected positive culture (UPC) *C acnes* continues to be heavily researched.^9,10,14,15,16,17,18^ *C acnes* infections are characteristically difficult to diagnose because of their indolent nature, and they usually lack clinical signs common to many infective processes.^13,16^ In the shoulder arthroplasty literature, standard infectious laboratory markers, C-reactive protein (CRP) and erythrocyte sedimentation rate (ESR), were found to be elevated in only 10% of patients.^19^ To complicate the clinical picture further, *C acnes* contamination has been reported between 7% and 13%.^15,20^ Criteria for the diagnosis of a true *C acnes* infection has been suggested by Lutz et al. and Asseray et al.^17,18^ Our study uses similar criteria, combining clinical features in the presence of one positive culture.

Although there is a growing recognition of the effect of *C acnes* in the general orthopaedic population, the literature involving a wide range of orthopaedic patients is lacking. The diagnosis of a true *C acnes* infection versus an indeterminate culture-positive patient continues to be a topic of interest and controversy given its frequent isolation in deep tissue infections.^16,17,21^ We conducted a retrospective study aiming to determine the clinical significance of a single sterile culture-positive *C acnes* sample after an open orthopaedic surgery case. We hypothesized that in patients with a positive *C acnes* culture, in the setting of no clinical or laboratory signs of infection, does not correlate with true postoperative infection. We also hypothesized that patients who fail to meet the diagnostic criteria of true infection do not require antibiotic therapy.

## Methods

### Patients

A retrospective cohort study was conducted at the Cedar Sinai Medical Center, a tertiary trauma care center (886 beds), from January 1, 2013, to May 1, 2020. International Review Board committee approval was received before beginning the investigation. All patients who were surgically treated by the primary surgeon (E.B.) were identified through the hospital computer electronic medical record database. Any patient with at least one positive culture for *C acnes*, in any intraoperative orthopaedic tissue sample, was included in this study. Relevant patient data were extracted from medical records. There were no exclusion criteria. All antibiotics used were reviewed and characterized by purpose (prophylactic or therapeutic), antibiotic class, route of administration, and duration. All orthopaedic surgeries were reviewed and characterized by anatomical site and type.

Clinical records were reviewed for patient medical and surgical history. Information collected included radiographic, laboratory, microbiologic data, along with surgical, treatment, and follow-up information. For the purpose of this study, postoperative antibiotic administration was classified as “antibiotic prophylaxis” when less than 24 hours of antibiotics was administered and as “antibiotic treatment” when treatment lasted more than 24 hours.

Before incision, patients were prepared and draped according to standard hospital protocols, which involve thorough washing of the surgical site with 70% isopropyl alcohol. The surgical site is then prepared in an aseptic manner with 2% chlorhexidine gluconate in 70% isopropyl alcohol (ChloraPrep; Becton, Dickinson and Company) in two layers. After sterile draping, an adhesive antimicrobial clear drape (Ioban; 3M) was placed over the surgical site. In addition, if working near the shoulder, the axilla was isolated by means of adhesive drapes.

All culture swabs were obtained from the deep tissue in a sterile fashion without making contact to the epidermis or dermal layer of the skin. It should be noted that the skin knife was discarded from the surgical field after initial skin incision. The primary surgeon ensured sterility was maintained, avoiding any contact with the superficial structures, to obtain each intraoperative culture. All orthopaedic specimens were transported under anaerobic conditions and inoculated on routine bacteriological media, including a pre-reduced CDC anaerobic blood agar (CDC) and chopped meat medium broth. Incubation in anaerobic conditions was done for a minimum of 14 days. *C acnes* was identified either by rapid identification (gram-positive coryneform rods, positive 15% catalase, and positive indole spot test) or by routine culture.

We examined all patients with positive intraoperative cultures obtained during open orthopaedic surgery. Suspected true *C acnes* infection criteria used were one positive culture with associated elevated laboratory markers (CRP, ESR, and white blood cell count) and/or clinical infection manifestations (local inflammatory signs [swelling, erythema, and pain], implant loosening, nonunion, and bone erosion). Patients with a positive *C acnes* culture and none of the above findings were classified into an indeterminate group (subclinical infection or contaminant). *C acnes* monoinfection was defined as an infection solely caused by *C acnes*. A coinfection was defined as an infection caused by ≥2 microorganisms both of which were considered clinically significant.^16-18,22^

### Statistical Analysis

Nonparametric analyses were conducted. The relationship between a true *C acnes* infection and interval levels of measurement (ie, age and body mass index) were analyzed using the Wilcoxon rank sum test. The relationship between a true *C acnes* infection and nominal levels of measurement (ie, ethnicity) was analyzed using the chi square test. A *P*-value of 0.05 or less was considered to be statistically significant. Statistical analyses were conducted using IBS SPSS Statistics 26.

## Results

### Description of the Studied Cohort

During the study period, 48 patients matched our inclusion and exclusion criteria (Table 1). There were 31 male and 17 female patients. The mean age of patients with a suspected *C acnes* infection was 57 years, and the mean age for patients in the indeterminate group was 55 years. The ethnicity of the study participants was 36 White, three Hispanic, three African American, three Asian, and three Middle Eastern.

**Table 1:** Comparison of Those Who met the Criteria for a C acnes Infection Versus Those Within the Indeterminate Group

	*C acnes* Infection (*n* = 8)	Did not meet *C acnes* Infection Criteria (*n* = 40)	*P*-Value
	Mean (SD)	Mean (SD)	
Age, yr	57.13 (16.89)	54.78 (18.51)	0.75
BMI	25.66 (4.23)	27.87 (6.39)	0.37
	Frequency (%)	Frequency (%)	
Sex			0.50
Female (n = 17)	2 (11.76%)	15 (88.23%)	
Male (n = 31)	6 (19.35%)	25 (80.64%)	
Ethnicity			0.11
White (n = 36)	5 (13.89%)	31 (86.11%)	
Hispanic (n = 3)	0 (0%)	3 (100%)	
Black (n = 3)	2 (66.67%)	1 (33.33%)	
Asian (n = 3)	1 (33.33%)	2 (66.67%)	
Middle Eastern (n = 3)	0 (0%)	3 (100%)	
Surgery type			1.00
With implants (n = 6)	1 (16.67%)	5 (83.33%)	
Without implants (n = 42)	7 (16.67%)	35 (83.33%)	
Location of surgery			0.76
Shoulder (n = 15)	2 (13.33%)	13 (86.67%)	
Hip (n = 12)	4 (33.33%)	8 (66.67%)	
Arm (n = 6)	1 (16.67%)	5 (83.33%)	
Thigh (n = 6)	0 (0%)	6 (100%)	
Back (n = 4)	1 (25%)	3 (75%)	
Hand (n = 1)	0 (0%)	1 (100%)	
Neck (n = 1)	0 (0%)	1 (100%)	
Immunosuppressed			0.07
Yes (n = 12)	4 (33.3%)	8 (66.67%)	
No (=38)	4 (10.5%)	32 (84.21%)	
Previous surgery on the surgical site			0.04*
Yes (n = 11)	4 (36.36%)	7 (63.63%)	
No (n = 37)	4 (10.81%)	33 (89.18%)	
Previous biopsy on the surgical site			0.79
Yes (n = 22)	4 (18.2%)	18 (81.8%)	
No (n = 26)	4 (15.4%)	22 (84.6%)	
Additional antibiotics before surgery			0.00*
Yes (n = 3)	3 (100%)	0 (0%)	
No (n = 45)	5 (11.1%)	40 (88.9%)	
Clinical postoperative symptoms of infection			0.00*
Yes (n = 8)	8 (100.0%)	0 (0%)	
No (n = 40)	0 (0%)	40 (100.0%)	

Altogether, the cases included 14 lipoma excisions, 10 soft-tissue sarcoma resections, 2 implant removals, 2 elastofibromas, 2 neurogenic tumor excisions, revision joint arthroplasty, impending long bone fracture fixation, enchondroma protuberans excision, osteochondroma excision, chondrosarcoma resection, desmoid tumor excision, enchondroma curettage, chondroblastoma curettage, dermatofibrosarcoma protuberans, bone cyst excision and curettage, metastatic renal cell carcinoma resection, pigmented villonodular synovitis excision, hip arthroplasty, and myxoma excision. Antimicrobial prophylaxis received at the time of the surgery consisted mainly of a first-generation cephalosporin (n = 46, 96%). The average follow-up period was of 11.5 months (range 8 to 101 months).

#### True *C acnes* Infection Cohort

**Figure 1:**
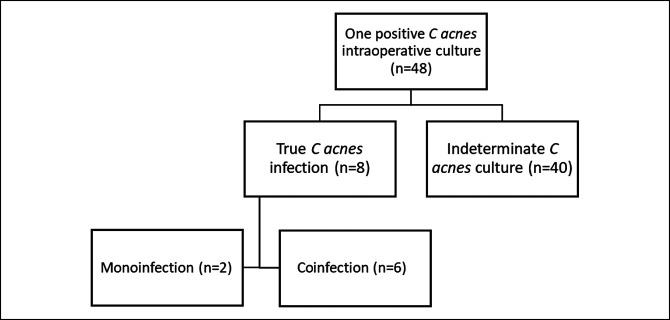
Patient flowchart.

According to our definition, *C acnes* was considered to be a causative pathogen in eight patients who had at least one positive *C acnes* culture, whereas the other 40 culture-positive patients were considered indeterminate (subclinical infection or contaminated) by *C acnes*. In the subset of true *C acnes* infections, two were monoinfections and six were coinfections with an additional organism (Figure 1). The additional organisms identified included *Staphylococcus aureus*, *Staphylococcus lugdunensis,* Group B *Streptococcus,* and *Peptostreptococcus*.

The true *C acnes* infections occurred in patients undergoing the following procedures: two thigh sarcoma resections, two lipoma resections, two groin sarcoma resections, one total hip revision, and one implant removal. In the true infection group, six of the eight patients underwent repeat surgery for irrigation and débridement for infection treatment. All patients who underwent a second surgery for infection treatment were classified as coinfections. The two true *C acnes* monoinfections were in patients undergoing removal of implants and lipoma excision. Both were treated with a short course (10 days) of doxycycline and required no additional surgical intervention.

The postoperative complications identified in the true infection group of patients included revision total hip arthroplasty due to coinfection of *S aureus* and *C acnes*, cellulitis after lipoma resection that subsequently resolved with oral antibiotics, and abscess formation after soft-tissue sarcoma and lipoma resections.

Local inflammatory signs were present in 6 of the 8 patients (75%) meeting the criteria for true *C acnes* infections. The white blood cell count, CRP, and ESR were elevated in only 2 of the 8 patients with true *C acnes* infection (25%). Of the two true monoinfections, one patient showed local incisional cellulitis postoperatively; however, neither patient had elevated inflammatory laboratory findings.

#### Comparison of True *C acnes* Infection and Indeterminate (Subclinical Infection or Contaminant) Cohorts

##### Implants

In total, 42 surgeries were conducted with orthopaedic implants that included either implantation or removal of implant. In both cohorts, a total of six procedures (12.5%) included orthopaedic implants. In patients classified as suspected true *C acnes* infection, 1 of the 8 patients (12.5%) underwent surgery that included orthopaedic implants. In the indeterminate group, 5 of the 40 patients (12.5%) underwent a procedure with implants involved. No notable difference was found between the two groups.

##### Location

Surgery was conducted in the following locations: shoulder (15), hip (12), arm (6), thigh (6), back (4), hand (1), and neck (1). In patients with a true *C acnes* infection, the most frequent locations were the hip and shoulder, four and two patients, respectively. Overall, in patients with positive *C acnes* culture from the hip, 4 of the 12 patients (33.33%) resulted in a suspected true infection while 8 of the 12 patients (66.67%) resulted in indeterminate classification. Similarly, with the location of the surgery involving the shoulder, 2 of the 15 patients (13.33%) had a suspected true infection while 13 of the 15 patients (86.67%) were classified as indeterminate. No notable difference was identified between the two cohorts.

##### Immunosuppressed

Twelve patients met the criteria for the immunosuppressed cohort, 33.3% of whom were diagnosed with a true infection. The criteria included history of receiving chemotherapy or radiation, chronic steroid use, immunodeficiency disorders, and taking immunosuppressive drugs. No notable difference was identified between the two cohorts.

##### Previous surgery on the surgical site

History of surgery on the same surgical site was found to be statistically significant for an increase in true *C acnes* infection classification (*P* = 0.04). Eleven of the 48 patients had previous surgical intervention on the same surgical site. Of those who had undergone previous surgery, 4 of 11 patients (36.36%) fit the criteria for a true *C acnes* infection. By contrast, 4 of 37 patients (10.81%) who did not have any documented history of prior surgery met the criteria for a true *C acnes* infection.

##### Previous biopsy on the surgical site

A total of 22 of the 48 patients (45.83%) had undergone previous CT-guided biopsy on the surgical site for preoperative oncologic diagnosis and surgical planning. No statistically significant correlation was observed between previous biopsy and true *C acnes* infection diagnosis. Of the eight patients classified as true *C acnes* infection, 4 of the 8 (50%) had a previously done biopsy.

##### Additional antibiotics before surgery

Patients with a history of antibiotics use before surgery had a statistically significant increase in suspected true *C acnes* infections (*P* < 0.001). All three patients who received preoperative antibiotics were diagnosed with a true *C acnes* infection. In patients who did not receive preoperative antibiotics, 5 of 45 (11.1%) were diagnosed with a suspected *C acnes* infection.

##### Postoperative antibiotic use

Patients receiving postoperative antibiotics were all within the true *C acnes* infection cohort. Postoperative antibiotics included Zosyn, cefepime, Keflex, Bactrim, Levaquin, and cefazolin. No postoperative therapeutic antibiotics were given to the indeterminate culture-positive group.

##### Clinical postoperative symptoms of infection

As expected, patients with clinical signs and symptoms of infection were found to be more frequently classified as a true *C acnes* infection (*P* < 0.001). Eight of the eight patients (100%) who displayed the symptoms of postoperative infection (fever, erythema, swelling, and wound issues) were diagnosed with a true *C acnes* infection. By contrast, no patients in the indeterminate group showed clinical signs of infection. Postoperative laboratory markers were found to be elevated in only three patients in the entire cohort, two of whom were diagnosed with coinfections. One patient in the indeterminate group had a mildly elevated ESR without other clinical signs of infection.

## Discussion

The clinical significance, optimal treatment strategy, and outcomes in patients with positive intraoperative *C acnes* cultures and no overt signs of infection are largely debated.^8,12,23^ The incidence of UPC has been reported in the shoulder literature as high as 17%; however, only 5.9% to 12.1% of the UPC patient population went on to progress to a true infection postoperatively.^8,10,24^ In our cohort, the presence of a positive *C acnes* culture was associated with a diagnosis of true infection in 8 of the 48 cases (16.6%). Removing patients with coinfections, 2 of the 48 patients with UPC (4.2%) were diagnosed with a true *C acnes* monoinfection. The monoinfection patients were treated with a 10-day course of doxycycline with no further surgical treatment. 40 of the /48 patients (83.3%) in the indeterminate cohort were treated with close surveillance and no antibiotic therapy and required no additional surgical treatment.

Although an accepted definition of a *C acnes* infection remains a topic of debate, we used simplified criteria to define suspected true *C acnes* infection.^16-18^ One positive culture and perioperative clinical and/or laboratory signs of infection placed the patient in the true infection group. Our criteria was adapted from the guidelines of Asseray et al^18^ that had a diagnostic probability of >90% for a *C acnes* infection: ≥2 positive cultures in addition to one of the following criteria: perioperative findings, local signs of infection, ≥2 previous operations, or orthopaedic devices or one positive culture in addition to three of the following criteria among perioperative findings, local signs of infection, ≥2 previous surgical operations, orthopaedic devices, and inflammatory syndrome.

Skin flora contains many native bacteria. However, controversy remains where exactly *C acnes* inhabits.^14,25,26^ Lee et al^2^ revealed that *C acnes* is common on the epidermal surface of unprepared skin of normal patients and is found more frequently and in greater numbers in male patients with increased sebaceous glands. Their observations support the overarching concept that the epidermis, dermis, and hair follicles are potential contamination sources of the *C acnes* culture positivity.^12,14,20,27-29^

In the case of *C acnes* positivity in one or more deep surgical samples, the question for the surgeon is whether there is an infection, possible indeterminate infection, or sample contamination. Probable infection should be made based on associating bacteriological and clinical findings.^16-18^ In cases of indeterminate infection or suspected contamination, simple surveillance without antibiotic initiation has been argued in the literature.^9,10,17,21^ Grosso et al. retrospectively reviewed the results of 17 patients undergoing revision shoulder arthroplasty with at least one UPC who were not treated for infection. Their patients had no clinical symptoms or laboratory signs of infection. They found a low clinical recurrence rate (5.9%) of infection for this untreated revision arthroplasty group and concluded that prolonged antibiotic therapy may not be necessary.^10^ Similarly, Dramis et al^9^ identified 50 patients with prosthetic joints from whom *C acnes* was isolated at least once. Patients with UPC were treated with surveillance with no antibiotics. Only one patient had additional revision surgery for infection. Our findings support clinical surveillance with a close follow-up for 2 years in the UPC indeterminate group.

In the shoulder arthroplasty literature, Kim et al investigated treatment strategies regarding patients with an UPC, including *C acnes*, without overt signs of infection in revision shoulder arthroplasty. In their systematic review of 1402 patients undergoing revision shoulder arthroplasty, 16.7% of the patients had an UPC. Occurrence of a true infection from an UPC after revision shoulder arthroplasty was seen in 24 shoulders (10.2%). They concluded that there is a low risk of having a true infection from an UPC after revision shoulder arthroplasty without clinical signs of perioperative infection.^8^

Additional research evaluating C acnes UPCs in open shoulder surgery was conducted by Mook et al.^15^. Patients with a history of shoulder surgery or any concern for active or previous shoulder infection were excluded. In their study, three soft-tissue samples were obtained from the shoulder, as well as a sterile sponge. Overall, 20.5% of the surgeries yielded at least one specimen removed for culture that was positive for bacterial growth, and 13.0% of the sterile control sponge specimens had positive culture growth. *C acnes* represented 83.0% of all positive cultures. They identified male sex and preoperative corticosteroid injections as risk factors for bacterial growth on culture. They concluded that *C acnes* is isolated through culture at a substantial rate from clinically noninfected shoulders; however, there is a substantial level of culture contamination.^15^ Similarly, the infections in our cohort occurred mostly in male patients (5/8, 71.4%); however, they failed to reach significance.

A recent study by Lavergne et al examined the clinical difference between *C acnes* infection and contamination. A total of 68 patients had at least one positive *C acnes* culture, 35 of whom were considered to be infected. The infections were mostly found in males and located in the shoulder girdle. Ninty-one percent of the infections occurred at a site already containing an orthopaedic implant. Local inflammatory signs were present in half of the cases when an infection was diagnosed. Coinfection with other pathogens was present in 31% of the patients. This study demonstrates the importance of clinical correlation and the importance of accurate diagnosis of a *C acnes* infection.^16^ Our study seeks to further Lavergne et al work by applying a modified diagnostic protocol in patients where only one positive *C acnes* culture is found. Orthopaedic implants were involved in only 14.5% of our study group, which lacks power to compare directly with the work of Lavergne et al.

UPCs in patients without clinical signs or symptoms of infection continue to confuse the clinical picture and may lead to overdiagnosis and treatment. The rate of contamination from the native microbiome has been recorded ranging from 7% to 15% and has varied depending on the institution conducting this study.^14,15,20,27,28,29,30,31^ Currently, there is no definitive benchmark skin surface preparation proven to prevent inoculation of bacteria from the epidermal and dermal structures into the deep tissues at the time of the skin incision; however, hydrogen peroxide shows promise.^14,20^ Additional research into preoperative skin preparation may aid in decreasing culture contamination and provide clarity to the *C acnes* UPC picture. The *C acnes* UPC conflict demonstrates the importance of clinically correlating a positive culture in an otherwise healthy patient.^17,18^ Which patients go on to a clinical infection seems to depend on the size of inoculum, suitability of the microenvironment for growth, relative proportions of various pathogenic strains, and the host response to *Cutibacterium*.^7,28^ Other important factors to consider when examining the risk of a *C acnes* infection include male patients who previously underwent surgical intervention and implant-associated procedures.^3,8,15,16^

In our study, the presence of a positive *C acnes* culture was associated with a diagnosis of true infection in only 17% of the cases. The indeterminate patients truly encompass the definition of an UPC cohort. The patients in this group failed to show any clinical or laboratory signs of infection perioperatively and did not require additional postoperative antibiotics or surgical treatment. Close clinical monitoring of these patients was found to be the appropriate management. We did not find any notable true infection association between sex, ethnicity, immunosuppression, and surgery location. Interestingly, we also failed to find any statistically significant connection between previous CT-guided biopsy and diagnosis of a true *C acnes* infection. This suggests that the sterile biopsy may not increase the risk of future *C acnes* culture positivity and infection. Our results add to the existing literature that patients who underwent previous surgery on the planned surgical site are at an increased risk of developing a true *C acnes* infection. In addition, we found that antibiotic use before surgery and clinical signs of infection postoperatively were associated with the diagnosis of a true *C acnes* infection.

Our study has several limitations. It is retrospective, and complete data may not have been available for all patients. We identified suspected true *C acnes* infections as those patients with at least one positive culture and one perioperative infectious sign. It could be argued that the second positive culture is necessary to confirm the suspected infection. The arthroplasty literature recommends three tissue samples be sent for culture to improve the yield and to decrease the likelihood of a false-negative or positive culture.^32^

In patients with an UPC, we did not differentiate between contaminant and subclinical *C acnes* colonization. Increasing the number of cultures and having a control arm would have aided in separating the two groups. We lacked notable power with many categories, including orthopaedic implant. Although our study may allow us to apply the conclusions to a general orthopaedic practice involving soft-tissue procedures, we are unable to conclude on implant-related *C acnes* positive cultures.

To decrease selection bias, we did not exclude any cases. The patients were identified directly from the hospital database, and collection of data was done by two independent evaluators. There was a wide range of follow-up length for each patient, which did not allow for consistent long-term follow-up data on all patients. This limits our findings to applying to only short-term outcomes. Future long-term studies with a sterile control group and an increased number of sterile culture swabs per patient should be undertaken to identify the containment group from the indeterminate culture-positive patients. Additional prospective multicenter studies with increased patient numbers are necessary to better define the optimal treatment management strategy.

## Conclusion

Orthopaedic surgeons often encounter positive cultures obtained from presumably uninfected cases during the course of their career. We found that a single positive *C acnes* culture rarely represents a true infection in patients who lack clinical signs of infection. Given the inherently high false-positive rate with long-hold cultures, clinical correlation in patients presenting with culture-positive *C acnes* is recommended. In this study, we found that patients undergoing open orthopaedic surgery with at least one positive *C acnes* intraoperative culture, in the absence of clinical or laboratory findings suggesting infection, have a low likelihood of requiring treatment with antibiotics or revision surgery. By contrast, patients who present with *C acnes* coinfections underwent previous surgery on the surgical site or those who display clinical signs of postoperative infection should be treated with organism-specific antibiotics.
